# A systematic review of interventions targeting modifiable factors that impact dietary intake in athletes

**DOI:** 10.1017/S0007114523001769

**Published:** 2024-01-28

**Authors:** Amy Janiczak, Rebekah Alcock, Adrienne Forsyth, Gina Louise Trakman

**Affiliations:** 1 Sport, Performance and Nutrition Research Group, Department of Sport, Exercise and Nutrition Sciences, La Trobe University, Melbourne, VIC, Australia; 2 Essendon Football Club, Melbourne, VIC, Australia; 3 School of Behavioural and Health Sciences, Australian Catholic University, Fitzroy, VIC, Australia

**Keywords:** Interventions, Sport, Diet, Dietary impacts, Nutrition

## Abstract

Appropriate dietary intake has been found to positively impact athletes’ performance, body composition and recovery from exercise. Strategies to optimise dietary intake often involve targeting one or more of the many factors that are known to influence dietary intake. This review aims to investigate the types and effectiveness of interventions used to impact modifiable factors of dietary intake in athletes. MEDLINE, CINAHL, SPORTDiscus and Web of Science were searched from inception to May 2022 for intervention studies that measured dietary intake with a quantitative tool and explored at least one factor thought to influence the dietary intake of adult athletes. Study quality was assessed using the ADA Quality Criteria Checklist: Primary Research. Twenty-four studies were included. The most common interventions focused on nutrition education (*n* 10), macronutrient adjustment (*n* 7) and physical activity (*n* 5). The three most common factors thought to influence dietary intake addressed were nutrition knowledge (*n* 12), hunger and appetite (*n* 8), and body composition (*n* 4). Significant changes in dietary intake were found in sixteen studies, with nutrition education interventions returning significant results in the largest proportion of studies (*n* 8). Study quality within this review was mostly average (*n* 4 < 50 %, *n* 19 50–80 %, *n* 1 > 80 %). As studies included were published between 1992 and 2021, interventions and factors explored in older studies may require up-to-date research to investigate possible differences in results due to time-related confounders.

Good nutrition supports athletes’ health, performance, and recovery from training and competition^(1)^. Adjusting dietary intake to meet sports nutrition recommendations has been found to improve outcomes such as body composition (increased muscle mass^(2)^), competitive edge in performance (VO^2^ max^(3)^, handgrip strength^(4)^ and sprint performance^(5–7)^) and post-exercise recovery (carbohydrate improves glycogen resynthesis after endurance activities^(8)^, protein increases muscle protein synthesis and decreases muscle degradation^(9)^, and adequate energy and macronutrients reduce risk of injury^(10)^). However, athletes often do not follow consensus dietary recommendations. In this context, it is important for nutrition professionals working with athletes to understand ways in which dietary intake can be modified.

Nutrition education interventions have been used to influence athletes’ dietary behaviours^(11)^. Nutrition interventions have included a broad range of topics, such as energy, macronutrient, micronutrient and hydration principles, as well as meal frequency and timing, and supplement use^(12)^. In relation, multiple interventions in athletes have included behavioural strategies, such as enablement, training, environmental restructuring, modelling and coercion^(13)^. Terenzio et al.^(11)^ identified positive impacts on consumption of vegetables, nuts, legumes and fish (*P* < 0·05, *P* < 0·001, *P* < 0·001 and *P* < 0·05, respectively), while Boidin et al.^(12)^ reported inconsistent changes in dietary intake across the studies included in the review. A systematic review by Bentley et al.^(13)^ identified that the majority of interventions with a focus on behavioural strategies had a positive impact on improved dietary behaviours, made possible through several approaches.

Nutrition knowledge is one factor that is often targeted for change in nutrition education interventions^(14,15)^, with a systematic review finding that nutrition education interventions are efficacious at improving nutrition knowledge^(15)^. Good nutrition knowledge is one of many factors that impact dietary intake^(16–18)^. Several frameworks have been established to operationalise factors that influence diet intake. In athletes, Birkenhead and Slater^(16)^ identified physiological and biological; lifestyles, beliefs, and knowledge; psychological; social; and economic, and Pelly et al.^(17)^ identified similar factors. Likewise, in the general population DONE^(18)^ noted biological, demographic, psychological, situational, social, cultural, product, micro, meso/macro, industry and government factors. In this context, it is relevant to assess other factors that may have an impact. For this review, the factors focused on are hedonic hunger, nutrition knowledge, macronutrient contribution to total energy intake, and body image and weight control. These have been identified by previous literature as factors within the Birkenhead and Slater framework^(19)^ able to be modified by nutrition professionals. By focusing on elements that may be more easily modified by nutrition professionals, this review will explore factors most suitable to modification through interventions identified.

Hedonic hunger is associated with susceptibility to environment food cues^(20)^ or the desire to eat for the pleasure of the taste^(16)^. A recent study has found high risk of orthorexia and higher levels of hedonic hunger within individuals engaged in sport at any level than sedentary individuals^(21)^.

Nutrition knowledge has been shown to have a positive relationship with improved dietary behaviours^(22)^. Previous research has identified that athletes with a higher level of nutrition knowledge often exhibit more positive dietary behaviours^(14)^. Studies exploring the improvement of nutrition knowledge often rely upon nutrition education interventions^(23,24)^.

Macronutrient contribution to total energy intake has been shown to have an impact on total energy intake, with increased protein intake increasing satiety and therefore reducing total energy intake^(25)^. Athletes’ dietary intake may modify macronutrient contribution to total energy intake for purposes of periodisation^(1,26)^. Studies into macronutrient contribution to total energy intake in athletes have largely explored intake similarity to current guidelines^(27,28)^ and impact on performance^(29–32)^.

Body image and weight control are linked due to sports’ requirements for athletes to meet body composition goals or ideals and the methods used to meet those goals^(16,33)^. Studies have found that athletes within sports which recommend weight control have a higher prevalence of dissatisfaction with body image, as well as increased risk factors for eating disorders^(34)^ and aesthetic sport athletes were found to have a higher level of body dissatisfaction than endurance, fitness and weight class athletes^(35)^.

While nutrition education interventions are common, and the impact of these on athlete’s dietary intake^(12)^ and improved nutrition knowledge^(15)^ have been previously reviewed, there is a dearth of studies on the impact of other modifiable factors in athletes, and results of these studies are yet to be synthesised. A systematic review by Boidin et al.^(12)^ found that fourteen of the twenty-two included studies reported significant change in at least one aspect of dietary intake due to a nutrition education intervention. Similarly, Tam et al.^(15)^ reported a significant nutrition knowledge increase due to nutrition education interventions that were largely face-to-face delivered. The exploration of these interventions and their impact on both modifiable factors and dietary intake is necessary to inform effective design for future interventions. Therefore, the purpose of this review is to investigate the types and effectiveness of interventions used to impact the factors of hedonic hunger, nutrition knowledge, macronutrient contribution to total energy intake, and body image and weight control which influence dietary intake in athletes.

## Methodology

### Materials and methods

This systematic review was conducted in accordance with PRISMA guidelines^(36)^ and prospectively registered with PROSPERO (protocol registration ID 2022 CRD42022342781).

### Search method and information sources

Search strategy was developed by AJ with support from an academic librarian, which was then reviewed by GT, AF and RA. This was designed to capture information on food intake and factors assessed in our previous work^(37)^. One reviewer (AJ) systematically searched MEDLINE, CINAHL, SPORTDiscus and Web of Science databases from database inception up to and including 18 May 2022, with initial searches conducted 29 March 2022. Search terms included subject headings of diet, eating, nutritional sciences, dietetics, sports nutritional sciences, and athletes, and keywords of ‘diet* intake’, food, ‘feeding behavio?r’, ‘eating behavio?r’, ‘energy intake’, ‘nutrition knowledge’, ‘sport* nutrition knowledge’, ‘hedonic hunger’, appetite, compensat*, ‘macronutrient balance’, ‘high protein’, ‘high carbohydrate’, ‘high fat’, ‘body composition’, ‘weight control’, ‘body image’, ‘mak* weight’, ‘nutrition science’, ‘modifiable factor’, athlete*, and sport*. Limits for English language and journal articles were used. Full MEDLINE search strategy is available in Supplementary Material.

### Eligibility criteria

In order to be included in this review, studies were required to fulfil eligibility criteria as outlined in Table 1. Athletes were defined as any individual competing, participating or performing in a sport. For the purposes of this review, elite military personnel were considered athletes and were included within the review, although reserve military personnel were excluded. Recreational athletes participating in local sporting leagues were required to participate in a minimum of 2·5 h of exercise per week^(38–40)^. Adolescent athletes (less than 18 years of age) were excluded from this review due to the potential for age-related confounding factors and differences in dietary intake during growth^(1,41)^. If papers included a mixed cohort that reported adolescent and adult results separate, the results from the adult group would be included.

**Table 1.** Inclusion and exclusion criteria

PICO and study design	Inclusion criteria	Exclusion criteria
Study type	Original research. Quantitative research (RCT, quasi-experimental study and before–after study). English language.	Reviews Qualitative research Grey literature Case studies with *n* 1 Cross-sectional study
Population	Athlete populations (recreational, elite, sub-elite and college athletes within any sport). Adults (18+ years of age).	Non-healthy individuals
Intervention	Must include a factor that influences dietary intake. Must include an intervention that examines dietary intake and influences a modifiable factor to change dietary intake.	
Outcome	Must measure dietary intake through some quantitative measure.	

Dietary intake could include diet quality score, measure of habitual dietary intake, energy intake or nutrient intake. PICO, Population, Intervention, Comparator, and Outcome; RCT, randomised controlled trials.

### Screening process

**Fig. 1.**
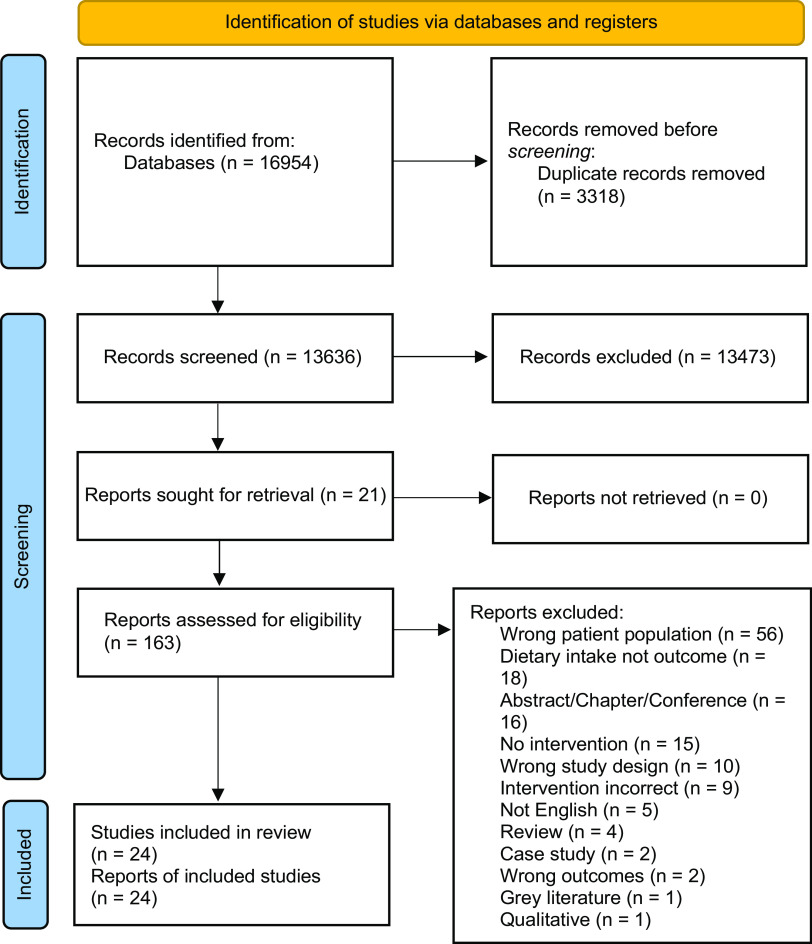
PRISMA flow chart for screening process. Source: Page MJ, McKenzie JE, Bossuyt PM, Boutron I, Hoffmann TC, Mulrow CD, et al. The PRISMA 2020 statement: an updated guideline for reporting systematic reviews. BMJ 2021;372:n71. doi: 10.1136/bmj.n7.

All records were imported to Covidence^(42)^, where duplicates were removed, and screening was subsequently completed. Two reviewers (AJ and GT or AF or RA) independently screened all papers for eligibility by reviewing title and abstract. Articles were requested for retrieval through the university library, with all articles being retrieved. Eligible articles were then screened in duplicate (AJ and GT or AF or RA) for full-text review (Fig. 1). Reasons for exclusion were noted within Covidence at the time of exclusion using customised exclusion categories. Disagreements were screened by a reviewer (GT or AF or RA) that was not involved in the original screening.

### Data extraction and data items

Covidence was used to extract data from the included studies using a pre-designed, purpose-built extraction form. Two reviewers (AJ and GT, AF, or RA) independently extracted the data from all included studies. Inconsistencies in extracted data were first discussed to attempt to reach consensus; if consensus could not be reached, a third reviewer (who was not involved in the initial extraction) was enlisted to decide. Data extracted included author and date, key study information (aim, location (country), setting (university or laboratory details), study design and recruitment methods), intervention information (intervention type and details), the factor being investigated (factor, measurement tool and results), dietary intake (measurement tool used and results), participant demographics (sample size, age and sex), and correlation measures between factor being investigated and dietary intake where available.

### Quality assessment

The quality of individual studies was assessed using Quality Criteria Checklist: Primary Research^(43)^, for the assessment of all studies. The process and guidelines for quality assessment were agreed upon among researchers prior to assessment commencement. Points were allocated for each question within the scale – ‘yes’ accounting for one point and ‘no’ or unclear receiving zero points for that question.

### Analysis

Narrative synthesis was used to analyse the data collected due to the heterogeneity of methods, and outcomes measured did not support meta-analysis.

## Results

### Included studies

Initial data searches provided 16 954 studies that were imported into Covidence; after duplicates (*n* 3318) were removed, 13 636 articles remained for title/abstract screening. The title/abstract screening stage excluded a further 13 473 papers, leading to inclusion of 163 papers for full-text screening. A total of 136 papers were excluded for a variety of reasons (available in Fig. 1). Twenty-four papers were included in the final extraction for this review (see Table 2).

**Table 2.** Data extraction table for dietary intake and factors being investigated

Study information	Population information	Study aim	Intervention type	Factor being investigated	Dietary intake	Results Experimental group		
Author names Year Location	Number Sex Age Sport/athlete type		Intervention Comparator	Factor Tool	Tool	Factor ↑/↓	Dietary intake ↑/↓	Correlation between dietary intake and modifiable factor
Abood et al., 2004^(53)^ USA	*n* 30 Female (100 %) 19·6 ± 1·1 (experimental), 19·4 ± 1·2 control Soccer/swim	To evaluate the benefits of nutrition education related to (a) changes in knowledge and (b) self-efficacy to make healthful choices, and (c) improved dietary intake.	I: 8-week cognitive theory-informed group programme. C: Scheduled, supervised study hall.	F: Nutrition knowledge and self-efficacy T: Nutrition knowledge measurement tool and self-efficacy measurement tool developed by authors	3-d diet record	Nutrition knowledge ↑* Self-efficacy ↑*	Total kcal - CHO - Fat - PRO -	N/A
Antonio et al., 2016^(44)^ USA	*n* 14 Male (100 %) 26·3 ± 3·9 years Resistance-trained	To examine the effects of high-protein consumption in a group of resistance-trained young males over 1 year.	I: High-protein diet (> 3 g/kg/d) C: Normal-protein diet (habitual intake)	F: Body composition T: Calibrated scale (anthropometry and weight), BODPOD (body densitometry)	Electronic food record (3 d per week for 1 year)	Weight (kg) ↑ Fat mass (kg) ↑ FFM (kg) - Body fat (%) ↑	Energy ↑*	N/A
Ballard et al., 2009^(56)^ USA	*n* 21 Male (100 %) 20 ± 1·8 Recreational weightlifting	To examine the effect of resistance exercise, with and without at least partial energy replacement using a CHO beverage, on plasma ghrelin, glucose, insulin, hunger and post-exercise energy intake.	I: CHO beverage consumption before acute resistance exercise. C: Placebo beverage consumption before acute resistance exercise and placebo beverage consumption with no exercise	F: Appetite T: Visual analogue scale (VAS)	Direct observation (of food consumed at a buffet, corrected for energy consumed in carbohydrate beverage)	Hunger -	Energy -	N/A
Barr & Costill, 1992^(66)^ USA	*n* 24 Male (100 %) 19·4 ± 0·4 years Swim	To assess the effect of a period of increasing training on dietary quantity and quality.	I: Two weeks double training (44 000 m/week) C: Usual training	F: Body composition T: Skinfolds	2-d food intake records	Skinfolds -	Energy (mid-season) ↑* CHO (mid-season) ↑* PRO - Fat (mid-season between group) ↑*	N/A
Buffington et al., 2016^(54)^ USA	*n* 153 Female (100 %) 18–27 years Air Force Academy	To evaluate the effects of an energy balance educational intervention and the COPE (Creating Opportunities for Personal Empowerment) cognitive behavioural therapy (CBT)-based intervention on the body composition, energy balance knowledge, stress, anxiety and nutrition intake of Div 1 US Air Force Academy female athletes.	I: Energy balance educational intervention + CBT-based intervention (a) The combined energy balance educational intervention with the COPE CBT-based intervention (E1), and (b) the COPE intervention alone (E2) C: A control group.	F: Nutrition knowledge, body composition, stress and anxiety T: Energy balance knowledge written test, DXA, self-report of perceived stress and GAD-7	24-h food recall	Nutrition knowledge ↑* Body fat(%) ↓* Stress ↓ Anxiety ↓	Intervention (knowledge + COPE) %kcal CHO ↑* %kcal PRO ↓ %kcal fat ↓* Intervention (COPE) %kcal CHO ↑ %kcal PRO ↑ %kcal fat -	N/A
Coccia et al., 2020^(23)^ USA	*n* 50 Male *n* 11, female *n* 39 19·62 ± 1·483 Varsity athletic team	To assess the feasibility and efficacy of a social media-based nutrition intervention on nutrition knowledge, dietary practices, BMI, self-efficacy and social support among NCAA Division I student-athletes.	I: Twitter nutrition knowledge intervention C: N/A	F: Nutrition knowledge and self-efficacy T: Nutrition knowledge questionnaire (NKQ) developed by authors and self-efficacy survey	The sixteen-item National Cancer Institute Fat Screener	Nutrition knowledge ↑* Self-efficacy ↑	% kcal from fat ↓* Fruit and vegetable intake ↑	N/A
Collison et al., 1996^(46)^ USA	*n* 100 (49 athletes) commenced, 41 (18 athletes) completed Female (100 %) Athletes 19·4 ± 1·2 years Non-athletes 21·1 ± 2·1 years Varsity athletic team	To determine the nutrition knowledge and attitudes of female university athletes and non-athletes; to implement a nutrition education programme and evaluate the impact on the knowledge, attitudes, and selected nutrient consumption of female university athletes in comparison with a group of university female non-athletes; and to determine satisfaction with body image for all of the women in the study.	I: Nutrition education programme C: N/A	F: Nutrition knowledge T: NKQ adapted from Werblow et al. (1978)	3-d diet records (2 weekdays and one weekend day)	Nutrition knowledge (pre- to post-test and pre-test to retention) ↑*	Energy ↓ P-value not provided	N/A
Durguerian et al., 2016^(55)^ France	*n* 11 Not stated Group 1:18·8 ± 0·8; Group 2:19·8 ± 0·7 Weightlifting	To mimic a typical major weightlifting championship framework to evaluate the effect of the nutritional strategy used by the competitors to lose weight on the performance and psychological responses just before the competition.	I: Diet group – lose about an average of 5 % of their body weight through self-determined means during 6 d. C: Habitual diet and weight maintenance.	F: Weight control, mood and stress T: Skinfolds POMS and REST-Q	6-d food record	Body weight ↓* BMI ↓ Body fat (%) ↓* FFM ↓* Total mood disturbance ↑ Global Recovery Score ↓* (Weight loss intervention group pre-test *v*. Post-test for all values)	Total energy ↓* CHO (g/kg) ↓* PRO (g/kg) ↓* Fat (g/kg) ↓*	N/A
Elias et al., 2018^(47)^ Malaysia	*n* 105 Male (100 %) Experimental group: 18·69 (sd = 0·88) Control Group: 23·26 (sd = 3·8) Team sport	To determine the effects of sports nutrition education intervention on KAP regarding sports-related nutrition and dietary intake among Malaysian elite team sports athletes.	I: 7-week nutrition education programme (food and healthy nutrition, macronutrient, micronutrient, fluid and hydration, nutrition before, during after training or competition, energy balance and weight management, dietary supplements) C: No education	F: Nutrition knowledge T: KAP questionnaire	3-d food record	Nutrition knowledge ↑*	Differences between experimental and control groups Energy ↑* CHO (g/kg) ↑* PRO (g/kg) ↑ Total fat (g) ↑*	N/A
Holliday and Blannin, 2017^(64)^ UK	*n* 12 Male (100 %) 21 ± 2 Endurance sport	To address the effect of exercise duration on subjective appetite, food intake, and circulating concentrations of acylated ghrelin, total PYY and total GLP-1 in trained endurance athletes, utilizing high-intensity exercise bouts, akin to the habitual training of endurance athlete, lasting 15, 30 and 45 min.	I: Different durations of intense aerobic exercise (15 min, 30 min and 45 min) C: N/A	F: Appetite T: VAS (adapted from Hill and Blundell)	*Ad libitum* test meal.	Appetite -	CHO (% energy) ↑ Fat (% energy) ↑ PRO (% energy) -	Energy intake correlation with VAS. (*r* < |0·5|, *P* > 0·1)
Kojima et al., 2016^(65)^ Japan	*n* 23 Male (100 %) 20 ± 0·3 Long-distance running	To investigate the time course of changes in appetite-related hormonal responses and spontaneous energy intake after a 20-km outdoor run (approximately 78 min in duration) in trained long-distance runners.	I: Physical activity intervention C: Identical period of rest	F: Appetite T: VAS	*Ad libitum* buffet meal	Appetite (experimental *v*. control) ↓* (0 and 15 min)	(Experimental *v*. control) Total energy ↓* PRO (% energy) ↓ Fat (% energy) ↓* CHO (% energy) ↑*	N/A
Kojima et al., 2018^(67)^ Japan	*n* 12 Male (100 %) 20·5 ± 1·1 years Track and field	To investigate the effect of WBC on post-exercise appetite regulation.	I: Whole-body cryotherapy C: Rest	F: Appetite and temperature T: VAS and NT logger monitor N543	*Ad libitum* buffet meal	Hunger (Time point *v*. pre) ↓* (0 and 15 min) Skin temperature ↓* (0, 15 and 30 min)	Energy intake (Experimental *v*. control) ↑* PRO - Fat ↑ CHO ↓	N/A
MacKenzie-Shalders et al., 2015^(57)^ Australia	*n* 10 Male (100 %) 21·2 ± 2·3 years Rugby union, rugby league, touch football or soccer	To assess the impact of a manipulation of protein dose (20, 40, 60 and 80 g) in a liquid whey protein supplement on subjective sensations of appetite and food intake in a cohort of concurrently training athletes who consume protein in excess of current recommendations.	I: Whey protein supplement (20, 40, 60 or 80 g protein) C: N/A	F: Appetite T: VAS	*Ad libitum* energy intake	Appetite (Between four groups) -	Dietary intake (Between four groups) CHO - Energy intake - Fat - Na -	N/A
MacKenzie-Shalders et al., 2016^(58)^ Australia	*n* 30 Male (100 %) 20·1 ± 1·4 Rugby	To manipulate dietary protein distribution through a protein supplement protocol and subsequently to assess the role of protein distribution and dietary energy intake on chronic gains in lean mass during a rugby preseason.	I: Ready to drink protein between meals at least 1 h from main meals C: Ready to drink protein with meals	F: Body composition T: Digital scales, DXA.	24-h recalls during each 6-week condition	Body composition Body weight - Lean mass - Fat mass -	Dietary intake Energy - Protein - Fat - CHO -	Correlation between average total energy intake and total change in lean mass (*r* = 0·43, *P* = 0·04)*
Martinelli, 2013^(48)^ Not stated	*n* 7 Female = 4, male = 3 18–23 years University elite sport	To evaluate the benefits of a nutrition education programme delivered by a qualified sports nutrition professional related to changes in knowledge, self-efficacy to make healthful choices and improved dietary intake.	I: Education programme C: N/A	F: Nutrition knowledge T: NKQ	7-d food record	Nutrition knowledge ↑*	Energy intake ↓ CHO intake - Fat intake ↓ PRO intake -	N/A
Melby et al., 2002^(59)^ USA	*n* 13 Female (100 %) 23·0 ± 0·8 Endurance sport	To investigate the effects of consuming a carbohydrate beverage *v*. placebo during moderate-intensity, long-duration exercise on exercise and post-exercise fat and carbohydrate oxidation, and on post-exercise energy and macronutrient intake.	I: Carbohydrate beverage and physical activity intervention Moderate-intensity exercise and carbohydrate beverage; rest and carbohydrate beverage; moderate-intensity exercise and non-energetic placebo beverage C: Rest and non-energetic placebo beverage	F: Appetite T: VAS	*Ad libitum* buffet meal	Appetite -	Energy intake ↑ (exercise conditions *v*. non-exercise conditions) ↓* (CHO conditions *v*. no CHO conditions) CHO ↓* (CHO conditions *v*. no CHO conditions) Fat - Pro ↓* (CHO conditions *v*. no CHO conditions)	N/A
Monteyne et al., 2018^(60)^ UK	*n* 15 Male (100 %) 21 ± 1 years Resistance training	To compare drinks containing dextrose (i.e. carbohydrate) and whey protein consumed after resistance exercise on subsequent appetite and energy intake.	I: Whey protein supplement C: Dextrose (carbohydrate)	F: Appetite T: VAS	*Ad libitum* meal	Main effects of trial: Hunger - Fullness - DTE - PFC -	Energy intake ↓* (PRO *v*. CHO supplement)	N/A
Nascimento et al., 2016^(24)^ Brazil	*n* 32 - 21 adolescents and 11 adults Adolescents = 6 female, 15 male Adults = 11 males Adolescents 15·4 ± 0·35 Adults 23·7 ± 0·53 Not stated	To evaluate and compare the effect of a nutritional intervention in athlete’s body composition, eating behaviour an nutritional knowledge and to compare the effect of the nutritional intervention between adult and adolescent athletes	I: Nutrition education intervention – consultations on eating habits and athlete routine C: N/A	F: Nutrition knowledge and body composition T: Nutritional knowledge test (based on the studies of Gonçalves and Zawila, Steib and Hoogenboom) Anthropometric measures (stadiometer, electronic scales and skinfold calliper)	24-h recall + photo album	Nutrition knowledge ↑* Body mass (kg) ↑* BMI ↑ MAMC Adult: - Adolescent ↑* Fat mass (%) Adult: ↑ Adolescent: ↓ Lean mass (kg) - Fat (kg) ↑ (Change from pre- to post- intervention scores; changes in both groups unless otherwise specified)	Individuals consuming adequate serves pre-intervention Cereals ↓* Fruits ↓* Vegetables ↓* Meats/eggs ↑ Dairy products ↓ Beans/nuts ↑* Fats/oils ↑* Sweets ↑*	N/A
Rastmanesh et al., 2007^(49)^ Iran	*n* 72 Not stated 30 ± 7·6 years Not stated (athletes with physical disabilities)	To assess the nutritional knowledge and attitudes of athletes with physical disabilities and their coaches before and after nutrition education.	I: Nutrition education programme C: Usual training methods given by coach	F: Nutrition knowledge T: Adaptation of Zawila et al. NKQ	3-d food record	Nutrition knowledge ↑*	Energy intake (pre-test *v*. post-test) ↓* Ca intake (pre-test *v*. post-test) ↑*	N/A
Roberts et al., 2019^(61)^ UK	*n* 16 Male = 9, Female = 7 28 ± 2 Resistance training	To compare the satiating effect of two diets with a different protein content in resistance-trained subjects in energy deficit, participant well-being and training motivation.	I: Dietary protein intervention A moderately high-protein diet (PROMOD) with an intake corresponding to the recommendation for maximal performance (1·8 g/kg/d) and a very high-protein diet (PROHIGH) with a protein intake of 2·9 g/kg/d were used as main interventions. C: N/A	F: Appetite T: VAS	Diet record (duration not stated) and *ad libitum* meal	Hunger - Satisfaction ↑* (High-protein diet 5–7 d *v*. moderate-protein diet 5–7 d) Fullness - Desire to eat - Cravings ↑* (Moderate-protein diet −7 d *v*. Moderate-protein diet 1–3 d)	Energy intake ↑ Protein intake - CHO intake ↑ Fat intake ↑	N/A
Rossi et al., 2017^(45)^ USA	*n* 30: intervention group = 15, control group = 15 Male (100 %) Intervention = 19·3 ± 1·0 Control = 19·8 ± 1·4 Baseball	To investigate the effects of a brief SNEI on nutritional knowledge, nutrition status, body composition and performance during a 12-week offseason in NCAA Division I baseball players.	I: Nutrition education programme C: No education	F: Nutrition knowledge, macronutrient intake, energy intake, body composition T: Sports Nutrition Questionnaire (Reilly and Maughan, 2007) Standard anthropometry, calibrated scale and BODPOD	3-d food diary	Nutrition knowledge ↑* Weight ↑ Body fat (%) ↓* (intervention group significantly different from control group) FFM ↑ Fat mass (kg) ↓* (intervention group significantly different from control group)	Pre- to post-intervention changes Energy intake ↑* PRO intake ↑* CHO intake ↑ Fat intake ↑*	N/A
Valliant et al., 2012^(50)^ USA	*n* 11 completed (13 recruited) Female (100 %) 19·5 ± 1·0 Volleyball	To conduct an evaluation of dietary intake, nutrition knowledge, and whether education improves dietary intake of collegiate female volleyball players.	I: Education programme C: N/A	F: Nutrition knowledge T: Sports Nutrition Questionnaire (Reilly and Maughan, 2007)	3-d food diary	Nutrition knowledge ↑* Pre- to post-intervention	Energy intake ↑* CHO intake ↑* PRO intake ↑* Fat intake - Pre- to post-intervention	N/A
Yannakoulia et al., 2002^(51)^ Greece	*n* 32 Female (100 %) 20·5 ± 1·6 Dance	To implement and evaluate a nutrition intervention programme in a group of professional dance students.	I: Nutrition education programme C: N/A	F: Nutrition knowledge and body image T: NKQ, EAT-26, DEBQ, Weight Dissatisfaction and BDQ	3-d food records	Nutrition knowledge ↑* Body image EAT-26 Total ↓* DEBQ Total ↓ Weight Dissatisfaction ↓ BDQ - Pre- to post-intervention	Energy intake - Alcohol intake ↓*	N/A
Zaman et al., 2021^(52)^ Malaysia	*n* 21 Male = 52·4 %, Female = 47·6 % 23·8 ± 3·4 University sport	To determine the KAP level and as to evaluate the effectiveness of sports nutrition education on KAP and dietary intake among young university athletes.	I: Nutrition education programme C: N/A	F: Nutrition knowledge T: KAP-Sports nutrition questionnaire	3-d food record	Nutrition knowledge ↑* Pre- to post-intervention	Energy intake Male ↑* Female ↑* CHO intake (g) Male ↑* Female ↑* PRO intake (g) Male ↑* Female ↑* Fat intake (g) Male – Female ↑* Pre- to post-intervention	N/A

I, intervention(s); C, control; F, factor(s); T, tool(s); CHO, carbohydrate; PRO, protein; FFM, fat-free mass; %kcal, percentage of total energy intake in kilocalories; COPE, Creating Opportunities for Personal Empowerment; DXA, dual-energy X-ray absorptiometry; GAD- 7, General Anxiety Disorder-7; NCAA, National Collegiate Athletic Association; POMS, Profile of Mood States; REST-Q, Recovery-Stress Questionnaire; WBC, whole body cryotherapy; PYY, Peptide YY; GLP-1, glucagon-like peptide 1; KAP, Knowledge, Attitudes, and Practices; DTE, desire to eat; PFC, prospective food consumption; MAMC, mid-arm muscle circumference; EAT-26, Eating Attitudes test; DEBQ, Dutch Eating Behaviour Questionnaire; BDQ, Body Dissatisfaction Questionnaire; SNEI, sports nutrition education intervention.

Aims are as stated in the articles.

↑, increased in intervention group; ↓, decreased in intervention group; -, no change; N/A, not applicable.

* *P*-value < 0·05.

A total of 844 participants were included across all studies, with 454 male and 307 female participants (two studies did not identify sex). Six studies consisted of entirely female populations, while eleven were entirely male populations. The age of adult participants ranged between 18·69+/–0·88 and 30+/–7·6 years. Participants included were from a variety of populations, as follows: endurance athletes (*n* 2), university athletes (*n* 10), resistance-trained athletes (*n* 5), team sport athletes (*n* 2), dancers (*n* 1), and athletes with spinal cord injury and amputations (*n* 1), as well as three studies with unclear sport/exercise types. Nine countries were represented within this sample, as follows: USA (*n* 10), UK (*n* 3), Japan (*n* 2), Australia (*n* 2), Malaysia (*n* 2), Greece (*n* 1), Iran (*n* 1), France (*n* 1) and Brazil (*n* 1), with country unreported in one study. Six of the included studies included ethnicity information for the athletes, with four of these studies occurring within the USA (ethnicities included Caucasian, African American, Hispanic, Pacific Islander, Asian and other) and two within Malaysia (ethnicities included Malay, Indian, Chinese and Others).

The study designs included quasi-experimental (*n* 9), randomised crossover trial (*n* 7), randomised controlled trial (*n* 4), counter-balanced (*n* 1), randomised counter-balanced design (*n* 1), randomised double-blind trial (*n* 1) and randomised, double-blind, placebo-controlled trial (*n* 1). The types of interventions employed by researchers included nutrition education (*n* 10), macronutrient adjustment intervention (*n* 7), physical activity (*n* 5), cognitive theory-based intervention (*n* 2), weight loss (*n* 1) and whole-body cryotherapy (*n* 1). Eight factors were assessed within included papers: nutrition knowledge (*n* 12), hunger and appetite (*n* 8), body composition (*n* 4), macronutrient contribution to total energy intake (*n* 2), self-efficacy (*n* 2), body image (*n* 1), stress and anxiety (*n* 3), and use of weight control measures (*n* 1).

### Intervention effectiveness

Some papers report dietary intake (e.g. absolute macronutrient intake or diet quality) and macronutrient contribution to total energy intake which has crossover in results due to the fact that dietary intake and macronutrient contribution to total energy intake are often measured with the same tools. Where adjustment to macronutrient contribution to total energy intake were the focus of the intervention (e.g. a high-protein diet), macronutrient distribution has been reported separately to absolute macronutrient and other dietary intake^(44,45)^.

Nutrition education was the most used intervention among the included papers (*n* 10)^(23,24,45–52)^. Nutrition education interventions included twitter-based nutrition knowledge interventions, group education sessions (duration: between 2 weeks and 5 months), face-to-face education sessions and personalised individual consultations. In addition to dietary intake, all studies that employed a nutrition education intervention assessed nutrition knowledge, with body composition^(24,45)^, body image^(51)^ and self-efficacy^(23)^ also examined in some studies. The majority of nutrition education intervention studies utilised a quasi-experimental study design (*n* 8)^(23,24,45,46,48,50–52)^, with the two remaining studies using a randomised controlled trial study design^(47,49)^. Dietary intake was measured using 3-d food records (*n* 7)^(45–47,49–52)^, 7-d food records (*n* 1)^(48)^, 24-h recall with photo album (*n* 1)^(24)^ and a sixteen-item National Cancer Institute Fat Screener (*n* 1)^(23)^. These tools measured energy intake (*n* 8)^(45–52)^, macronutrient intake (*n* 5)^(45,47,48,50,52)^, Ca intake (*n* 1)^(49)^, alcohol intake (*n* 1)^(51)^, adequate intake of food groups (*n* 1)^(24)^, percentage of energy content from fat and fruit/vegetable intake (*n* 1)^(23)^. Nutrition knowledge was measured using nutrition knowledge scores from various tools (*n* 10)^(23,24,45–52)^, body composition was measured using standard anthropometry, calibrated scales, and BODPOD (*n* 1)^(45)^, as well as skinfold callipers^(24)^, self-efficacy was measured with a pre-validated tool to provide a composite score (*n* 1)^(23)^, and body image scores were calculated through EAT-26, DEBQ, and Weight Dissatisfaction surveys (*n* 1)^(51)^. In eight of ten studies that used a nutrition education intervention, dietary intake was positively impacted according to post-intervention testing with significant decreases in percentage of total energy content from fat^(23)^, alcohol intake^(51)^, Ca intake^(49)^, total energy intake (closer to recommendations)^(49)^ and sweets and oils^(24)^, as well as significant increases, closer to recommendations, in total energy intake^(45,47,50,52)^ and macronutrient contribution to total energy intake^(50)^. Non-significant changes were also noted in deceased total energy intake^(46,48)^, decreased % energy from fat^(48)^, increased consumption of meat and eggs and decreased dairy products consumption^(24)^, and increased fruit and vegetable intake^(23,47)^. Changes in factors being investigated for nutrition education interventions were largely significant. Nutrition knowledge in athletes significantly increased in all studies post-intervention compared with baseline^(23,24,45–52)^. There were significant changes in body composition, with reductions in body fat (%) and fat mass (kg) for the intervention group compared with the control group, while fat-free mass (kg) increased significantly for both intervention and control group between pre- and post-intervention measures^(45)^. Nascimento et al. reported significant increases in body mass (kg) for both adult and adolescent participants and a significant increase in mid-arm muscle circumference for adolescent participants between pre- and post-intervention measures, while other measures had non-significant changes^(24)^. Nutrition education interventions positively impacted body image scores. Yannakoulia et al. reported significant decreases in body image disturbance scores between pre- and post-intervention testing (EAT-26 total score and dieting subscale, DEBQ restraint subscale)^(51)^; however, this study did not include a control group. Mean self-efficacy scores increased from pre-intervention testing to post-intervention testing in the Coccia et al. study, which also did not include a control group^(23)^.

Two studies^(53,54)^ utilised a cognitive-based, theory-based intervention to influence nutrition knowledge (*n* 2), with Abood et al. also assessing self-efficacy^(53)^ and Buffington et al. also assessing body composition (*n* 1), stress (*n* 1), and anxiety (*n* 1)^(54)^. These cognitive-based, theory-based interventions have an integral component of self-efficacy to provide a sense of self-control in order to modify behaviour^(53)^ or teach participants coping mechanisms for managing stressful situations and improve emotional state and behaviours^(54)^. Both Abood et al.^(53)^ and Buffington et al.^(54)^ utilised a randomised controlled trial study design. Abood et al.^(53)^ used author developed surveys to measure nutrition knowledge and self-efficacy, while dietary intake was measured with an electronic food record. Buffington et al.^(54)^ used an energy balance knowledge written test to measure nutrition knowledge, DXA to measure body composition, self-report perceived stress and GAD-7 to measure stress and anxiety, respectively; dietary intake was measured using a 24-h food recall. Abood et al.^(53)^ noted a significant decrease in percent of total carbohydrate intake in the control group between pre- and post-testing. The intervention group of Abood et al.^(53)^ study demonstrated a significant increase in self-efficacy scores. Buffington et al.^(54)^ identified significant changes in percent of carbohydrates (increase) and percent of fat (decrease) consumed in the intervention group with an energy balance and a Creating Opportunities for Personal Empowerment (COPE) intervention each week, with significant changes not identified for the COPE intervention group, while there was a significant increase in percent of protein consumed in the control group. Significant increases in nutrition knowledge were reported for groups who participated in cognitive-based intervention groups^(53,54)^; however, Buffington et al.^(54)^ also found significant increases in nutrition knowledge within the control group, which participated in a supervised study session rather than the education sessions. Significant decreases in body fat percentage were noted within intervention groups, and stress scores significantly increased for control group without significant changes for intervention groups^(54)^. Buffington et al.^(54)^ found no significant changes in anxiety scores between pre- and post-intervention for any group.

A weight loss intervention was utilised to assess body composition, mood and stress in high-level weightlifters who are often required to reduce weight for competitions through a randomised counterbalanced trial^(55)^. The intervention group lost about an average of 5 % of their body weight through self-determined means during a 6-d period, while the control group consumed their habitual diet and maintained their weight. Dietary intake was measured using a 6-d food record, while body composition was measured through skinfolds, mood was measured through Profile of Mood States survey and stress was measured with the Recovery-Stress Questionnaire. Dietary intake of energy and all macronutrients was found to be significantly lower than consumption during the weight loss phase. Body fat percentage, body weight and fat-free mass were found to be significantly lower in the diet group than the weight maintenance group. Total mood disturbance increased in the weight loss intervention group from pre-intervention testing to post-intervention testing, while there was a significant reduction in the global recovery score for this group from pre- to post-intervention testing.

Macronutrient adjustment interventions were used in seven of the included twenty-four studies. Macronutrient adjustment interventions involved changing intake of one or more macronutrients via supplementation (*n* 5)^(56–60)^ or change in overall diet pattern (*n* 2)^(44,61)^. Macronutrient contribution to total energy intakes are considered factors to be investigated due to the possibility that throughout the course of the intervention, this factor may impact overall energy intake due to gastric emptying variation^(62)^ or changes in hormonal appetite response^(63)^. A macronutrient adjustment intervention was used to impact a variety of factors being investigated: hunger and appetite (*n* 5)^(56,57,59–61)^ and body composition (*n* 2)^(44,58)^. Studies that included a macronutrient adjustment interventions used a variety of study designs, such as randomised crossover trial (*n* 4)^(44,58,59,61)^, randomised double-blind trial (*n* 1)^(60)^, counterbalanced trial (*n* 1)^(57)^ and randomised, double-blind, placebo-controlled trial (*n* 1)^(56)^. Dietary intake was measured using a wide variety of tools, such as direct observation (*n* 1)^(56)^, 24-h recall (*n* 1)^(57)^, 7-d food record (*n* 1)^(58)^, *ad libitum* buffet meal (*n* 3)^(59–61)^, electronic food record (*n* 1)^(44)^ and diet record (*n* 1)^(61)^. Investigated factor outcomes were measured using a variety of tools, including visual analogue scale (VAS) for measuring appetite (*n* 4)^(56,59–61)^, nutrition knowledge questionnaire for measuring nutrition knowledge (*n* 1)^(58)^, digital scales (*n* 2)^(44,57)^, DXA (*n* 1)^(57)^ and BODPOD (*n* 1)^(44)^ for measuring body composition. Total energy intake *ad libitum* meal was significantly lower for those who consumed carbohydrate supplements before the meal than those who did not^(59)^, as well as those consuming protein supplementation *v*. carbohydrate supplementation^(60)^. However, Roberts et al.^(61)^ identified significant differences between moderate-protein and high-protein diet groups in *ad libitum* buffet consumption, with an increase in protein intake and decrease in carbohydrate intake in the high-protein diet intervention group. No significant differences were found in total energy intake for three of the included studies^(56–58)^. The Ballard et al.^(56)^ study evaluated the impact of carbohydrate supplementation *v*. a placebo beverage prior to acute resistance exercise. MacKenzie-Shalders et al.^(57)^ explored the impact of whey protein supplement dose variation (20, 40, 60 or 80 g of protein). MacKenzie-Shalders et al.^(58)^ investigated the consumption of protein supplementation as a between-meal supplement *v*. consumption of the protein supplementation with meals. Interventions exploring appetite in relation to macronutrient adjustment had mixed results, with some studies indicating no significant difference in appetite due to the intervention^(56,57,59–61)^ and some studies having a significant difference in appetite/satiety based on time^(60,61)^. Body composition was not significantly impacted by macronutrient adjustment^(44,58)^ in any of the studies that reported on this outcome. MacKenzie-Shalders et al. found a significant correlation between the average total energy intake and total change in lean mass in an intervention exploring protein supplementation timing^(58)^.

Five of the included studies evaluated the impact of physical activity on dietary intake and one or more factor that influences diet intake, such as appetite (*n* 4)^(56,59,64,65)^ and body composition (*n* 1)^(66)^. Two studies also included macronutrient adjustment through supplementation as part of the intervention^(56,59)^. Interventions utilised included increases in exercise between groups^(64,66)^ and some with a rest condition used as the control^(56,59)^, as well as a study utilising both increases in exercise and a rest control^(65)^. The outcomes measured across these studies were VAS to measure appetite ^(56,59,64,65)^ and skinfolds to measure body composition ^(66)^. Dietary intake was measured through *ad libitum* test meals in three studies ^(59,64,65)^, one study used 2-d food intake records ^(66)^ and one study used direct observation of food consumed at a buffet that was subsequently corrected for energy consumed within the carbohydrate beverage that was part of the intervention ^(56)^. The majority of studies using a physical activity intervention were randomised crossover trials (*n* 4) ^(56,59,64,65)^, with one quasi-experimental trial ^(66)^. Two studies identified compensatory eating occurred after exercise. A significantly higher energy intake and carbohydrate intakes were identified in groups completing a longer physical activity sessions compared with those completing shorter physical activity sessions ^(66)^, which was in line with increased energy and carbohydrate requirement for fuelling this increase in exercise as suggested by athlete nutrition guidelines ^(1)^. Energy, protein (g) and fat (%) intakes measured through *ad libitum* test meals were significantly lower in the exercise group *v*. the resting control group, while carbohydrate (%) intake was significantly higher ^(65)^. Three studies did not identify any significant differences in total energy intake between those who exercised and those who did not ^(56,59)^, as well those who exercised different amounts ^(64)^. Appetite was affected in all studies measured, shown to significantly differ between exercise and no exercise conditions ^(56,59,65)^ and a significant main effect for time, with appetite rising after exercise until the consumption of the *ad libitum* test meal ^(64)^. Barr and Costill found no significant differences in skinfold between groups performing longer and control training regimens ^(66)^. Holliday and Blannin found no significant correlations between dietary intake and measures of appetite (VAS) through a physical activity intervention with differing durations of intense aerobic exercise ^(64)^.

Kojima et al. ^(67)^ use a whole-body cryotherapy intervention compared with a control group in a randomised crossover trial which received no whole-body cryotherapy to investigate appetite and subsequent dietary intake. Dietary intake was measured by an *ad libitum* buffet meal, while appetite was measured using a VAS and temperature was measured using NT logger monitor N543. Dietary intake for the intervention group was found to be significantly higher than the control group that rested post-cycling protocol. Non-significant changes in macronutrient intakes included decreased carbohydrate intake and increased fat intake, while protein intake remained steady. Hunger was found to be significantly lower in the intervention group receiving whole-body cryotherapy at 0- and 15-min intervals post-exercise and post-*ad libitum* meal consumption.

### Quality assessment

Quality assessment scores range between 33·3 % and 90·0 % (See Table 3). One paper scored > 80 % ^(45)^, nineteen papers scored between 50 % and 80 %, and four papers scored < 50 %. The research question was mostly well defined across studies (*n* 21 of 24, 87·5 %). Participant selection was poorly described across the majority of studies (*n* 3 of 24, 12·5 %), with eligibility criteria or sampling method often not described and samples being unrepresentative of the population. Blinding was not carried out in the majority of studies (*n* 3 of 21, 19 %), which was largely noted as an issue due to the inability to blind participants to the type of intervention they were receiving. Withdrawals were identified within thirteen studies, a small number of studies discuss methods of handling withdrawals (*n* 5 of 13, 38·5). All papers within this review utilised valid measures for dietary intake.

**Table 3.** Quality assessment using Quality Criteria Checklist: Primary Research^(43)^

Study information	Q. 1	Q. 2	Q. 3	Q. 4	Q. 5	Q. 6	Q. 7	Q. 8	Q. 9	Q. 10	%
Topic of question	Research question	Participant selection	Comparable groups	Withdrawal handling procedure	Blinding	Intervention procedure	Valid outcomes	Statistical analysis	Conclusions	Funding bias unlikely	
Abood et al., 2004^(53)^	1	0	1	–	0	1	1	1	1	1	77·8
Antonio et al., 2016^(44)^	1	0	–	1	0	1	0	0	0	0	33·3
Ballard et al., 2009^(56)^	1	0	0	–	1	1	1	1	1	0	66·7
Barr & Castill, 1992^(66)^	1	0	0	0	0	1	1	1	1	1	60·0
Buffington et al., 2016^(54)^	1	1	0	–	0	1	1	0	0	1	55·6
Coccia et al., 2020^(23)^	1	0	–	1	–	1	0	1	1	1	75·0
Collison et al., 1996^(46)^	1	0	1	0	–	1	1	0	1	0	55·6
Durguerian et al., 2016^(55)^	1	0	1	–	0	1	1	0	1	1	66·7
Elias et al., 2018^(47)^	1	0	0	1	0	1	1	1	1	1	70·0
Holliday & Blannin, 2017^(64)^	1	1	1	–	0	1	1	1	0	1	77·8
Kojima et al., 2016^(65)^	1	0	–	–	0	1	1	0	1	1	62·5
Kojima et al., 2018^(67)^	0	0	–	0	0	1	1	1	0	1	44·4
MacKenzie-Shalders et al., 2015^(57)^	1	0	1	–	0	1	1	1	1	0	66·7
MacKenzie-Shalders et al., 2016^(58)^	1	0	–	0	0	1	1	1	1	1	66·7
Martinelli, 2013^(48)^	1	0	–	0	0	1	0	1	1	0	44·4
Melby et al., 2002^(59)^	0	0	–	–	1	1	1	0	1	1	62·5
Monteyne et al., 2018^(60)^	0	0	1	–	1	0	0	1	1	1	55·6
Nascimento et al., 2016^(24)^	1	0	–	0	0	1	1	1	0	1	55·6
Rastmanesh et al., 2007^(49)^	1	0	1	–	1	0	1	1	1	0	66·7
Roberts et al., 2019^(61)^	1	0	–	1	0	1	1	1	1	1	77·8
Rossi et al., 2017^(45)^	1	1	1	1	0	1	1	1	1	1	90·0
Valliant et al., 2012^(50)^	1	0	–	0	0	0	1	0	1	0	33·3
Yannakoulia et al., 2002^(51)^	1	0	–	0	–	1	1	1	1	0	62·5
Zaman et al., 2021^(52)^	1	0	–	–	0	0	1	1	0	1	50·0
	21 /24	3 /24	8 N/A 12 /12	5 N/A 11 /13	4 N/A 3 /21	20 /24	20 /24	17 /24	18 /24	16 /24	
	87·5	12·5	66·7	38·5	19·0	83·3	83·3	70·8	75·0	66·7	

Note. 1 = yes, 0 = no/unclear, - = question is not applicable to this study.

## Discussion

The purpose of this review was to investigate the types and effectiveness of interventions used to impact factors that nutrition professionals can target to influence the dietary intake of athletes. Of the twenty-four papers included within this review, it was found that more studies utilised a nutrition education intervention (*n* 10) than any other type of intervention. Similarly, the most common factor being investigated was nutrition knowledge (*n* 12), with three included studies that focused on nutrition knowledge exploring additional factors ^(23,45,51)^.

### Intervention impact on factor being investigated

The impact that interventions have on factors being investigated must be considered. The most common factor explored was nutrition knowledge (*n* 12), which was predominantly investigated using nutrition education interventions. Each study exploring nutrition knowledge (*n* 10) reported a significant increase in pre- and post-intervention scores ^(23,24,45–50,52)^. In addition to nutrition education intervention, cognitive theory-based interventions were used to impact nutrition knowledge in two of the included studies, with both studies reporting significant increases in nutrition knowledge scores in groups receiving the cognitive theory-based intervention^(53,54)^. As the cognitive theory-based interventions appear to be quite closely related to nutrition education interventions, it is possible that the commonalities of these interventions account for the ability of these two intervention types to positively impact nutrition knowledge. These results somewhat concur with Tam et al. ^(15)^ review of the effectiveness of education interventions designed to improve nutrition knowledge in athletes where the majority (30/32 studies) reported significant improvement in nutrition knowledge within athlete populations. It is not surprising that nutrition education improves nutrition knowledge, as they often target this factor within the education programme. Nutrition education interventions should remain a mainstay of interventions for improving nutrition knowledge, as they have proven effective in the past^(14,68)^. It is not possible here to comment on which aspect of nutrition education interventions is best, as the interventions included are widely varied and not well documented in research studies. It would be prudent for future research to investigate types of education interventions. It is recommended that interventions are more thoroughly described within articles. As this factor can be effectively modified, it is prudent to have nutrition knowledge be a focus or inclusion within interventions. An underexplored area is how improvements in nutrition knowledge translate to dietary intake and subsequent outcomes.

Hunger and appetite were factors explored in eight of the twenty-four included studies. Interventions related to modifying physical activity or macronutrient adjustment interventions were found to impact hunger results. This is unsurprising given that physical activity and macronutrient manipulation are thought to have a relationship with hunger levels. In contrast to the studies included in this review, a study into hunger and appetite in regularly exercising women, who did not meet the definition for athletes, found a significant reduction in hunger due to their standard exercise regimens^(69)^. It may be beneficial for future research to consider utilising programmes that are aimed at modifying athletes’ dietary intake to include components relevant to modifying macronutrient intake (through supplementation or overall diet pattern change), especially within athlete groups where hunger or appetite are concerns for meeting/exceeding energy intake requirements. A nutrition education intervention was not used within these studies to explore hunger and appetite. Further exploration of how hunger and appetite may be modified through nutrition education interventions is necessary, as these interventions have been shown to be effective in modifying nutrition knowledge and dietary intake. These education interventions may focus on methods of modifying hunger and appetite through dietary strategies. However, due to athletes participating in regular and rigorous physical activity, it may not be appropriate to explore the relationship between physical activity and hunger/appetite and subsequent dietary intake due to disruption or potential impact to the athlete’s regimen, so other strategies may be needed. A possible strategy for athletes from weight category sports experiencing increases in hunger due to exercise may be to provide strategies for high-volume, low-energy foods. Alternatively, if an athlete has high-energy needs and exercise has decreased appetite, strategies for low-volume, high-energy foods may be beneficial.

Body composition was explored within six of the twenty-four included studies^(24,44,45,54,58,66)^. The results for these studies were varied, with three studies using some form of nutrition education intervention showing significant differences in body composition outcomes^(24,45,54)^. These findings indicate that body composition may be effectively impacted through education interventions, which supports previous evidence of nutrition education interventions significantly improving body composition in athletes^(70)^. It is worth noting the included studies cover a wide variety of sports, which have different recommendations and requirements for body composition. For this reason, it would be beneficial for future research to include some exploration of body composition within different sports and player position for team sports. The use of specialised nutrition education programmes to empower athletes to safely make these changes would be beneficial long term, with the supervision of sports nutrition/dietetics professionals. By empowering and educating athletes, it may be possible to increase adherence to dietary recommendations and improve health outcomes after their athletic career.

Other factors that were explored within this review include macronutrient contribution to total energy intake, self-efficacy, body image, stress and anxiety, and use of weight control measures. However, these factors have an insufficient number of papers included to provide any conclusion as to the effectiveness of interventions in their modification. Further research is required for other factors that influence dietary intake to support any conclusions. As above, it may be beneficial to include these factors in investigations using a nutrition education intervention.

### Intervention impact on dietary intake

As well as exploring how interventions impacted each of the factors being investigated, it was also important to consider how dietary intake was impacted by each intervention. The most common intervention utilised was the nutrition education intervention (ten out of twenty-four studies), which resulted in significant positive changes in some measure of dietary intake in five of the ten studies^(23,45,47,50,52)^. A previous review of the impact of nutrition education interventions on dietary intake in athletes found that about two-thirds of studies (14/22 studies) had a significant change in at least one aspect of dietary intake per study, although these results were inconsistent^(12)^. The Spronk et al.^(22)^ review identified that the majority of studies (19/29 studies) had a weak, positive relationship between higher nutrition knowledge and positive dietary behaviours in the general population. A previous study in general population children found a significant change in fruit intake, but no other variable^(71)^. While interventions other than nutrition education have been explored within the included studies, the results of these studies indicate that there has not been such a significant impact on dietary intake through interventions other than nutrition education as through nutrition education intervention themselves or intervention types have not been utilised widely enough to draw conclusions about their effectiveness. The macronutrient adjustment intervention was included within this review based on the Birkenhead and Slater^(16)^ review, which noted that macronutrient intake imbalances may lead to increased total consumption. Two of the seven included studies^(44,60)^ which utilised a macronutrient adjustment intervention reported significant change in energy intake. While there is evidence that macronutrient composition of the diet can impact health outcomes^(16,72)^ and body composition^(73)^, this review calls into question whether alteration of macronutrient contribution to total energy intake in an *ad libitum* diet has a positive impact on dietary intake (energy and macronutrient intake) or has sufficient impact to be considered for intervention use. Adjustment to macronutrient intake in athletes has long been used to impact performance, with carbohydrate periodisation used for improved carbohydrate availability and performance during competition^(74,75)^, as well as protein consumption after exercise being adjusted to improve muscle protein synthesis^(1)^. Similarly, two of the five included studies^(65,66)^ using a physical activity intervention reported no significant change in dietary intake in the intervention group. The Donnelly et al.^(76)^ review in healthy adults identified that the majority of included studies showed no significant changes in energy or macronutrient contribution to total energy intake with increased physical activity.

### Correlation between dietary intake and factors being investigated

Only two of the included studies^(58,64)^ explored the relationship between dietary intake and their respective factors being investigated of appetite and body composition. This relationship between dietary intake and nutrition knowledge has been explored in cross-sectional studies^(14)^; however, it is not possible to demonstrate causality with this study design. Based on the data gathered within this review, it is not possible to comment on how this relationship between dietary intake and investigated factors is moderated. Due to this lack of evidence, further studies exploring this relationship are needed.

### Strengths and limitations

Strengths of this study include the use of multiple researchers to complete screening and data extraction processes, which reduced likelihood of reviewer bias. The use of a wide range of databases to complete searches allowed for a comprehensive view of available publications. The quality of this review was enriched by the use of previously established frameworks of factors that impact dietary intake in athletes. There have been no previous reviews that explored these factors being investigated and dietary intake in relation to the interventions used, making this a novel review. Some limitations of the methodology for this review were the exclusion of grey literature and non-English studies. In this review, we have chosen to focus on the factors identified as we were interested in factors that could be modified in interventions. However, other factors exist that could influence dietary intake in athletes ^(16,17)^

### Quality assessment

The majority of papers within this review (19/24 studies) had moderate scores between 50 and 80 %. Boidin et al.^(12)^ review found poor study quality within single-arm studies and fair study quality within double-arm studies for intervention studies exploring dietary intake and nutrition education in athletes, with a poor reporting of intervention compliance and dietary intake collection methods. A review by Sanchez-Diaz et al.^(70)^ exploring the impact of nutrition education interventions on eating habits, nutrition knowledge, body composition and physical performance found that majority of studies exploring these factors had poor-to-good methodological quality. The quality of studies within the current review may show a higher quality of papers as compared with previous reviews.

### Future direction

Future directions for study may focus on factors that have been identified as having less primary research into their impact on dietary intake, such as cognitive theory-based interventions, weight loss practices and whole-body cryotherapy. The data gathered within this review could be used to develop an intervention focused on impacting multiple factors that influence dietary intake. Creation of an intervention to improve outcomes for both factors investigated here and dietary intake would allow researchers to focus on factors that athletes believe are most necessary to improve their performance and experience. Nutrition education is common and has been found to be effective in modifying targeted factors and dietary intake; therefore, it would be beneficial to explore this intervention type further with additional factors. It is not possible to say what type of nutrition education is most effective, as there is a great deal of heterogeneity within previous interventions. Therefore, it would be useful to explore the effectiveness of elements of education programmes in future studies. This review identified that no previous studies (between 1992 and 2022) have engaged the athletes or club-based stakeholders in creation of interventions (specifically nutrition education interventions) ^(77)^; by following this process of co-design, stakeholders would provide feedback to better target education interventions and increase engagement. Therefore, it would be beneficial for future athlete studies to utilise a co-design method for creation of interventions, involving a variety of shareholders from athletes, coaching teams and nutrition/dietetic professionals.

### Conclusion

The aim of this review was to explore types and effectiveness of interventions used to influence factors that impact dietary intake and dietary intake in athletes. It was hypothesised that the results of this review would inform future intervention design. About half of the interventions led to improvements in dietary intake. Despite interventions having targeted factors theoretically proposed to influence dietary intake, dietary intake was unchanged in several of the studies included in this review. Nutrition education interventions are likely the most common as they are easy to implement. Future nutrition education interventions may need to focus on translating changes in dietary intake through increased nutrition knowledge. A framework around the creation and use of these interventions would be useful for consistency of delivery and replicability. It would be beneficial to incorporate practical or procedural knowledge within these interventions and to involve athletes in the development of interventions through co-design.

## Supplementary material

For supplementary material accompanying this paper visit https://doi.org/10.1017/S0007114523001769. click here to view supplementary material
